# Identification and Analysis of the *ZmGDS1* Gene Family in Maize

**DOI:** 10.3390/genes17040379

**Published:** 2026-03-26

**Authors:** Qi Wang, Lufei Zhao, Pengfei Chu

**Affiliations:** College of Agriculture and Biology, Liaocheng University, Liaocheng 252000, China; 17860260583@163.com

**Keywords:** *GDS1* gene family, maize, genome-wide analysis, phylogenetics, leaf senescence

## Abstract

**Background/Objectives:** The *Arabidopsis thaliana GDS1* (Growth, Development and Splicing 1) gene has recently been identified as a key regulator linking nitrate signaling to leaf senescence. However, a systematic analysis of the *GDS1* gene family in maize (*Zea mays* L.) is lacking. This study aims to identify and characterize the *ZmGDS1* gene family in maize, providing a foundation for functional studies on their roles in growth, development, and low-nitrogen-induced leaf senescence. **Methods:** Putative *ZmGDS1* family members were identified by searching the maize B73 reference genome using BLASTP (version 2.11.0+) and HMMER (version 3.4), with the Arabidopsis GDS1 protein sequence as the query. Candidate sequences were verified for the presence of the conserved zf-CCCH domain using NCBI CD-Search and SMART. Phylogenetic relationships, gene structures, conserved motifs, chromosomal distribution, collinearity, and promoter cis elements were comprehensively analyzed using MEGA 11, TBtools (version 1.098), MEME (version 5.5.9), and PlantCARE. Phylogenetic trees were constructed using the maximum likelihood (ML) method with the LG+G+I model and 1000 bootstrap replicates. **Results:** Thirteen *ZmGDS1* genes were identified, distributed unevenly across eight maize chromosomes. Phylogenetic analysis classified the ZmGDS1 proteins into four distinct groups (A–D), revealing a lineage-specific expansion in group D. While all members contained the conserved zf-CCCH domain, their motif compositions varied considerably; ZmGDS1.1 exhibited the most complex structure, whereas ZmGDS1.12 had the most simplified. Subcellular localization predictions indicated that most ZmGDS1 proteins are targeted to the nucleus, consistent with a potential role as transcription factors. Promoter analysis revealed an abundance of cis elements associated with light response, hormone signaling (methyl jasmonate, abscisic acid, auxin), and stress responses. Notably, phylogenetically related genes tended to share similar cis-element profiles. **Conclusions:** This genome-wide analysis provides the first characterization of the *ZmGDS1* gene family in maize. The observed structural conservation and diversity, together with regulatory elements linked to senescence-associated signals, support the hypothesis that *ZmGDS1* genes may contribute to leaf senescence, particularly under low-nitrogen conditions. These findings provide a basis for future functional validation studies.

## 1. Introduction

Maizeis the most widely cultivated cereal crop and has the highest total grain yield in China. Its annual sown area remains stable at over 40 million hectares, and its production accounts for approximately 40% of the nation’s total grain output, giving it an irreplaceable strategic position in ensuring national food security and supporting the development of animal husbandry [1]. Grain yield in maize mainly depends on the accumulation of photosynthetic products in the canopy after silking. As the primary organ for photosynthesis, leaves’ functional duration directly determines the extent and intensity of grain filling [2]. Leaf senescence is the final stage of leaf development, orchestrated by finely tuned genetic programs; it is accompanied by chloroplast degradation, a decline in photosynthetic function, and the remobilization and transport of nutrients such as nitrogen and phosphorus to the developing grains [3,4]. Timely senescence facilitates efficient nutrient recovery and redistribution. However, premature leaf senescence leads to a sharp decrease in photosynthetic area, insufficient grain filling, a marked reduction in 1000-kernel weight and kernel number per ear, and ultimately a yield loss of 20–30% [5]. Therefore, deciphering the initiation and regulatory mechanisms of maize leaf senescence and identifying key genes that delay senescence and prolong leaf functional duration are core scientific challenges for breeding high-yielding and stable maize varieties [6].

The initiation and progression of leaf senescence are regulated by both endogenous genetic programs and external environmental cues [7,8]. Abiotic stresses such as drought, high temperature, low light, pathogen infection, and nutrient deficiency can induce or accelerate senescence [9,10]. Among these, nitrogen deficiency is the most common environmental factor triggering leaf senescence in agricultural production. Nitrogen is an essential component of chlorophyll, proteins, and nucleic acids. Insufficient nitrogen supply in the soil directly accelerates chlorophyll degradation, reduces photosynthetic rates, and increases hydrolytic enzyme activity in leaves, promoting premature yellowing and senescence [11]. In maize, the period from silking to grain filling represents the peak of nitrogen uptake and is a critical window during which premature senescence is most likely to occur. If soil nitrogen is inadequate at this stage, the basal leaves turn yellow and senescence rapidly progresses upward [12], severely limiting photosynthetic product accumulation and grain filling.

Applying nitrogen fertilizer is the main agronomic practice to delay senescence and sustain photosynthesis. However, excessive use raises production costs and causes environmental problems such as water eutrophication, soil acidification, and higher greenhouse gas emissions [13]. Therefore, reducing nitrogen input while maintaining leaf function and yield has become a key challenge for sustainable maize production [14]. An effective strategy is to identify key genes that regulate low-nitrogen-induced senescence and use them to breed varieties with extended leaf function and improved nitrogen use efficiency [4].

Recent years have seen substantial progress in understanding the molecular regulation of leaf senescence in model plants. In Arabidopsis, several transcription factor families—including NAC, WRKY, bZIP, and MYB—are known to regulate leaf senescence [15,16]. However, how nitrogen signals integrate into senescence networks and which key genes mediate this integration remain central questions.

A significant breakthrough came with the identification of *GDS1* (Growth, Development and Splicing 1; *AT3G47120*) in Arabidopsis [17]. Isolated through a yeast one-hybrid screen with a nitrate-responsive promoter element, *GDS1* encodes a nucleus-localized transcription factor that links nitrate signaling to senescence. Loss-of-function gds1 mutants exhibit accelerated leaf senescence under low-nitrogen conditions, while *GDS1* overexpressors display delayed senescence, prolonged leaf functional duration, and enhanced nitrogen use efficiency. Mechanistically, GDS1 acts through a dual regulatory mode. Under sufficient nitrogen, it represses the senescence-promoting transcription factors PIF4 and PIF5. Under low-nitrogen stress, GDS1 is degraded by the APC/C complex, which relieves this repression and initiates senescence [17]. As a member of the CCCH-type zinc finger protein family, AtGDS1 is characterized by its involvement in RNA processing and its role as a molecular link between nitrate signaling and senescence [17]. Although CCCH proteins are known to have diverse roles in plant development and stress responses [18], the functional conservation and diversification of GDS1 homologs in crops such as maize remain largely unexplored.

In stark contrast to the rapidly advancing understanding of *GDS1* function in Arabidopsis, systematic identification and functional analysis of *GDS1* homologues in maize are still in their infancy. Maize, a typical C4 crop, has a genome far more complex than that of Arabidopsis, and gene families often undergo significant expansion via whole-genome duplication events [18,19]. Several fundamental questions remain unanswered: Are there functional homologues of Arabidopsis *GDS1* in maize? What is the copy number of the *GDS1* gene family in the maize genome? What are the evolutionary relationships, gene structures, conserved domains, and cis-regulatory elements of different family members, and do their tissue-specific expression patterns differ? The publication of the B73 reference genome and the continuous improvement of high-quality genome annotations provide powerful resources for the rapid identification of candidate gene families in maize based on homology searches [20]. Systematic identification of maize *GDS1* family members, along with analysis of their evolutionary characteristics and expression patterns, is the primary prerequisite for dissecting the potential functions of this family in regulating maize leaf senescence. It also serves as a crucial bridge connecting fundamental research in model plants to applied research in crop genetic improvement [21].

Based on the background described above, this study used the Arabidopsis GDS1 (AT3G47120) protein sequence as bait to search for homologous genes in the maize B73 reference genome via BLASTP. Using bioinformatic approaches, we systematically identified *GDS1* family members in maize and characterized their phylogenetic relationships, gene structures, conserved motifs, chromosomal distribution, and promoter cis-elements. CCCH-type zinc finger proteins, including GDS1, play diverse roles in plant development and stress responses; thus, their systematic identification is a critical first step toward understanding their function. Through these analyses, we aimed to describe evolutionary features and generate testable predictions about the biological functions of the *ZmGDS1* gene family, with a focus on their potential roles in regulating leaf senescence under low-nitrogen conditions. This work also provides candidate genes for future functional studies.

## 2. Materials and Methods

### 2.1. Identification of ZmGDS1 Genes in the Maize Genome

The GDS1 protein sequence of Arabidopsis (AT3G47120) was downloaded from The Arabidopsis Information Resource (TAIR) database (https://www.arabidopsis.org/ (accessed on 2 March 2026)) and used as a query for BLASTP (version 2.11.0+) searches against the maize protein database (Zm-B73-REFERENCE-NAM-5.0.55) with an E-value threshold of 1 × 10^−5^. Genomic sequence files (gff3, protein, coding sequence, and genome) for maize were downloaded from the Phytozome database (https://phytozome-next.jgi.doe.gov/ (accessed on 2 March 2026)). The hidden Markov model (HMM) profile of the zf-CCCH domain (Pfam: PF00642) was obtained from the Pfam database (https://www.ebi.ac.uk/interpro/ (accessed on 2 March 2026)) and used for HMMER (version 3.4) searches. The gathering cutoff (E-value ≤ 0.01) was used to ensure only high-confidence domain matches were retained. After integrating the results from BLASTP and HMMER searches, non-redundant protein sequences (Table S1) were submitted to the NCBI CD-Search (https://www.ncbi.nlm.nih.gov/Structure/cdd/wrpsb.cgi (accessed on 2 March 2026)) and SMART (https://smart.embl.de (accessed on 3 March 2026)) servers to confirm the presence of the conserved zf-CCCH domain. Proteins containing this domain were considered members of the maize GDS1 gene family. The identified genes were named *ZmGDS1.1* to *ZmGDS1.13* sequentially, following the order of chromosomes (from chromosome 1 to 10) and the ascending order of their physical positions on each chromosome.

### 2.2. Chromosomal Localization and Collinearity Analysis of ZmGDS1 Genes

Chromosomal positions of the *ZmGDS1* genes were retrieved from the maize genome annotation file. For collinearity analysis, pairwise BLASTP searches (E-value < 1 × 10^−10^) were performed to identify gene pairs within the maize genome. Based on the BLASTP results, chromosomal locations were visualized using TBtools(version 1.098).

### 2.3. Phylogenetic Analysis of ZmGDS1 Proteins

GDS1 protein sequences from Arabidopsis, wheat (*Triticum aestivum*), maize, rice (*Oryza sativa*), and sorghum (*Sorghum bicolor*) were aligned using the MUSCLE algorithm implemented in MEGA 11.0 software. A maximum likelihood (ML) phylogenetic tree was constructed using MEGA 11.0 with the LG+G+I model, which was selected as the best-fitting model according to the Bayesian Information Criterion (BIC). The analysis utilized pairwise deletion and 1000 bootstrap replicates to assess node support.

### 2.4. Analysis of Gene Structure, Conserved Motifs, and Promoter Cis-Acting Elements

The exon-intron structures of *ZmGDS1* genes were determined from the maize gff3 file and visualized using TBtools. The 2.0 kb upstream sequences from the start codon of each ZmGDS1 gene were extracted from the maize genome and used as promoter regions for cis-element analysis, a region generally considered sufficient to encompass core regulatory elements. Cis-acting elements were predicted using the PlantCARE website (http://bioinformatics.psb.ugent.be/webtools/plantcare/html/ (accessed on 7 March 2026)). The identified elements were categorized into hormone response, stress response, and other functional groups. Conserved motifs in ZmGDS1 proteins were identified using the MEME (Multiple Expectation Maximization for Motif Elicitation) online tool (version 5.5.9). The search was configured to identify up to 10 distinct motifs (Table S2), with an optimal motif width between 6 and 200 amino acids. The distribution of these motifs among family members was visualized using TBtools.

### 2.5. Prediction of Physicochemical Properties and Subcellular Localization

The physicochemical properties of the ZmGDS1 proteins, including the number of amino acids, molecular weight, theoretical isoelectric point (pI), instability index, aliphatic index, and grand average of hydropathicity (GRAVY), were calculated using the ExPASy ProtParam online tool (https://web.expasy.org/protparam/ (accessed on 18 March 2026)). To predict the subcellular localization of the ZmGDS1 proteins, the full-length amino acid sequences of the 13 identified members were submitted to the WoLF PSORT server (https://wolfpsort.hgc.jp/ (accessed on 18 March 2026)). WoLF PSORT predicts protein localization sites based on known sorting signals and amino acid composition. Predictions were also cross-validated using the Plant-mPLoc server (http://www.csbio.sjtu.edu.cn/bioinf/plant-multi/ (accessed on 18 March 2026)), which is specifically designed for plant proteins. The localization site with the highest score from WoLF PSORT was recorded, and consistency between the two tools was checked.

## 3. Results

### 3.1. Identification, Genome Distribution, and Collinearity Analysis of GDS1 Genes in Maize

**Figure 1:**
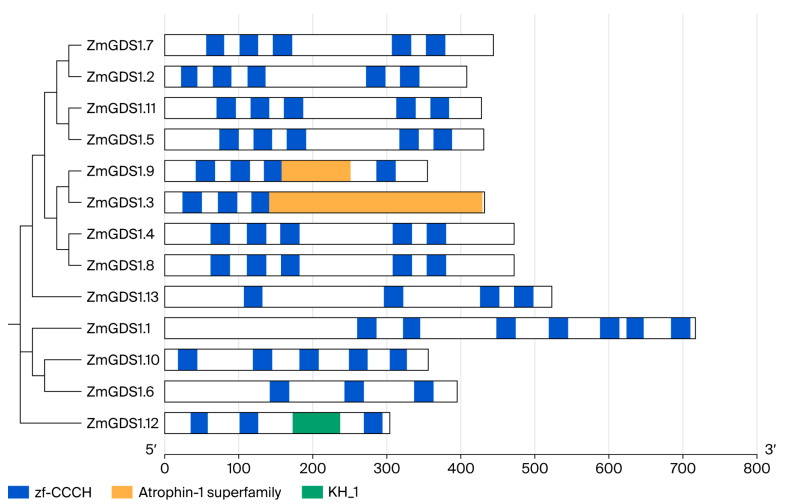
Conserved domain analysis of ZmGDS1 family members.

**Figure 2:**
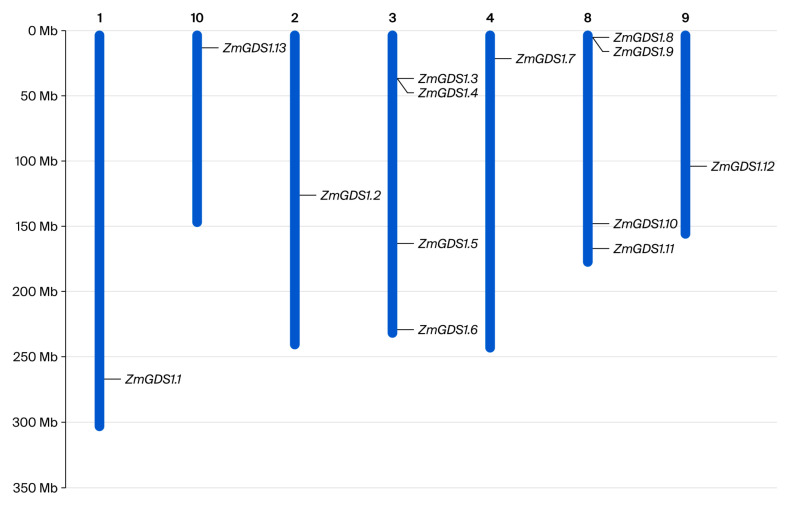
Chromosomal localization of the *ZmGDS1* gene family. The numbers above display the number of chromosome, while The image below the numbers displays the location of genes.

**Figure 3:**
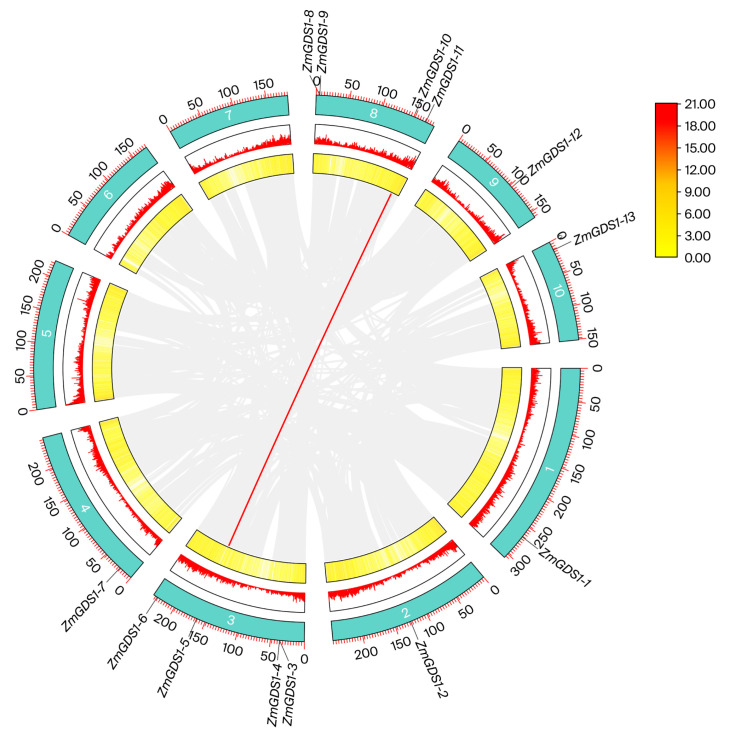
Genome distribution and collinearity analysis of the *ZmGDS1* family in maize. The cyan-green bands represent the 10 maize chromosomes, with the scale indicating physical position (Mb). The red peak diagram shows gene density/expression distribution, and the yellow ring indicates GC content distribution. Gray lines denote genomic collinearity blocks.

By integrating HMMER and BLASTP search results, we obtained 13 non-redundant protein sequences, all of which contained the zf-CCCH domain (Pfam: PF00642) (Figure 1) and were therefore designated as members of the maize *GDS1* family. Based on their chromosomal locations (Figure 2), the corresponding genes were named *ZmGDS1.1* to *ZmGDS1.13*. The naming followed the chromosome order (1–10) and the ascending order of gene start positions on each chromosome, as illustrated in Figure 2. A Circos diagram was generated to visualize the distribution and evolutionary relationships of the *ZmGDS1* gene family within the genome (Figure 3). The *ZmGDS1* genes were predominantly located in chromosome arm regions with high gene density and high GC content, a distribution pattern that closely matched the trends of the red peak map (gene density/expression level) and the yellow ring (GC content). This suggests that these genes reside in transcriptionally active and recombination-prone environments, a typical feature of functional genes in the genome. Collinear blocks within the maize genome (gray/white lines) revealed numerous homologous regions arising from whole-genome duplication events. Red-highlighted lines specifically marked a pair of *ZmGDS1* genes exhibiting a strong collinear relationship, indicating that they likely originated from a common ancestral gene through duplication and represent paralogous genes that have retained high sequence similarity during evolution.

### 3.2. Physicochemical Properties of Maize ZmGDS1 Proteins

The physicochemical properties of the ZmGDS1 proteins were characterized (Table 1). The number of amino acids ranged from 303 (ZmGDS1.12) to 716 (ZmGDS1.1), suggesting possible structural variations such as truncations or domain insertions/deletions among family members. The predicted molecular weights ranged from approximately 31.5 kDa (ZmGDS1.12) to 81.0 kDa (ZmGDS1.1). Theoretical isoelectric points (pI) varied from 5.55 (acidic) to 9.58 (basic), indicating potential differences in subcellular localization or interaction environments. With the exception of ZmGDS1.12 (instability index 37.98), all members had instability indices >40 and were predicted to be unstable proteins. The grand average of hydropathicity (GRAVY) values was negative for all members (ranging from −0.911 to −0.524), indicating that they are hydrophilic. Aliphatic indices were generally low (41.89–57.26), further supporting their hydrophilic nature.

Subcellular localizations of ZmGDS1 proteins were predicted using WoLF PSORT and Plant-mPLoc (Table 2). Most ZmGDS1 proteins were predicted to localize to the nucleus, with high WoLF PSORT confidence scores (e.g., ZmGDS1.3, ZmGDS1.4, ZmGDS1.7, and ZmGDS1.8 each scored 13.0 for nuclear localization). This nuclear localization aligns with the expected role of GDS1 proteins as transcription factors or RNA-binding proteins involved in nuclear processes such as transcriptional regulation and RNA splicing. A few members showed alternative or dual predictions: ZmGDS1.6 and ZmGDS1.10 had similar scores for nucleus and cytosol/mitochondria, suggesting possible dual targeting or dynamic distribution. Notably, ZmGDS1.13 was predicted by WoLF PSORT to localize primarily to chloroplasts (score 4.0), while Plant-mPLoc predicted nuclear localization, indicating that this member may have a distinct or additional function in plastids. These predictions provide a basis for future experimental validation of ZmGDS1 functions in different subcellular compartments.

### 3.3. Phylogenetic Analysis of GDS1 Genes

**Figure 4:**
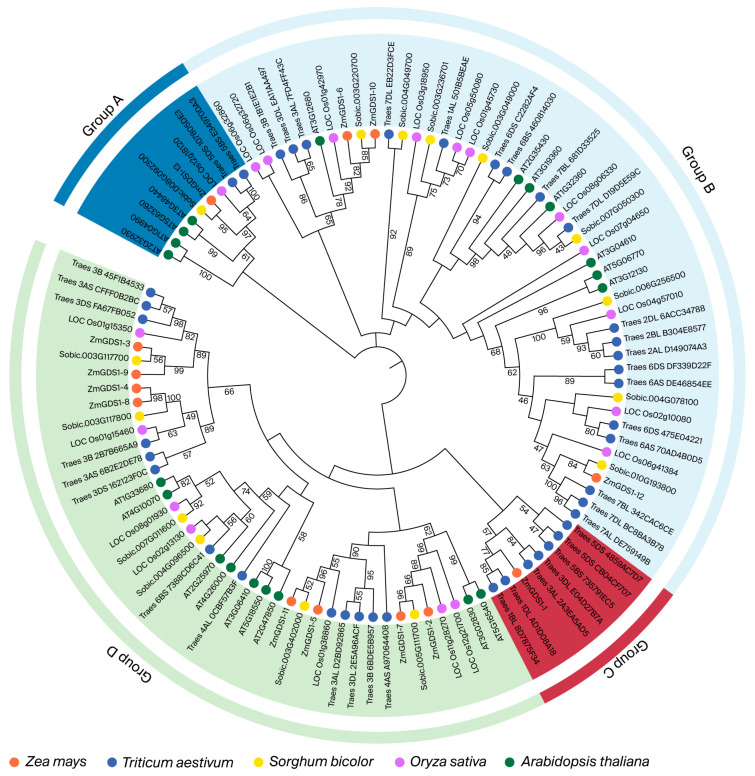
Phylogenetic tree of *GDS1* genes from Arabidopsis, wheat, maize, rice, and sorghum. The tree was constructed using the maximum likelihood (ML) method with the LG+G+I model and 1000 bootstrap replicates. Colors indicate species: Arabidopsis (green), wheat (blue), maize (red), rice (purple), sorghum (orange).

To elucidate the evolutionary relationships among *GDS1* genes, we constructed a phylogenetic tree using 107 GDS1 protein sequences from five species (Table S3): 20 from Arabidopsis, 15 from sorghum, 19 from rice, 40 from wheat, and 13 from maize (Figure 4). The analysis classified these proteins into four groups (A–D). The tree was constructed using the maximum likelihood method with the LG+G+I model and 1000 bootstrap replicates. Group B contained the largest number of members (48 sequences), including 3 ZmGDS1 proteins, 7 Arabidopsis proteins, 11 rice proteins, 19 wheat proteins, and 8 sorghum proteins. Group D was the second largest, comprising 42 members with 8 maize, 9 Arabidopsis, 13 wheat, 6 rice, and 6 sorghum sequences. Group A contained nine members: one ZmGDS1, four Arabidopsis, two wheat, one rice, and one sorghum sequence. Group C had the fewest members, with only eight protein sequences, including one ZmGDS1.

### 3.4. Gene Structure and Motif Composition of ZmGDS1 Genes

**Figure 5:**
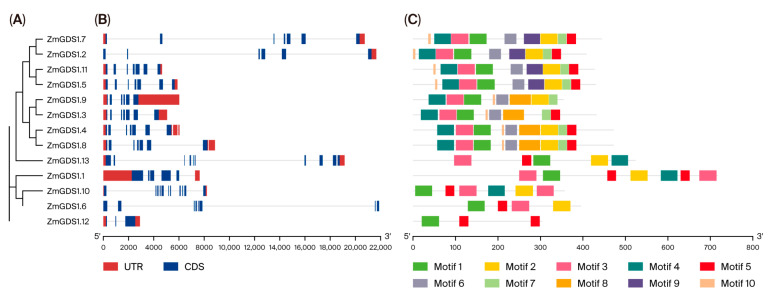
Phylogenetic relationships, gene structures, and conserved motif compositions of the *ZmGDS1* gene family in maize. (**A**) Phylogenetic tree, constructed using the maximum likelihood (ML) method with the LG+G+I model and 1000 bootstrap replicates. (**B**) Exon intron structures: yellow boxes represent CDS, black lines represent introns, and blue boxes represent UTRs. (**C**) Conserved motifs identified by MEME; different motifs are indicated by colored boxes.

To examine the structural characteristics of the *ZmGDS1* gene family, we compared the coding sequences (CDS) with their corresponding genomic DNA sequences to determine the arrangement of coding and non-coding regions. The results revealed both conservation and diversity in gene structure, with distinct features associated with evolutionary branches (Figure 5). Specifically, the number, length, and distribution of introns varied considerably among members of different branches. Genes with closer phylogenetic relationships (e.g., *ZmGDS1.7* and *ZmGDS1.2*; *ZmGDS1.11* and *ZmGDS1.5*) tended to share similar exon–intron patterns, whereas more distantly related members exhibited greater structural divergence. For instance, *ZmGDS1.9* had a relatively long continuous CDS region, whereas the CDS of *ZmGDS1.6* was more fragmented. Such structural differences may be associated with functional specialization. Furthermore, the distribution patterns of UTRs (red boxes) and CDSs (blue boxes) were highly conserved among closely related members, reflecting strong functional constraints during evolution. These structural findings provide key evidence supporting the classification and functional differentiation of the *ZmGDS1* family and lay the groundwork for future studies on transcriptional regulation and evolutionary mechanisms.

To explore conserved sequence features of ZmGDS1 proteins, we used the MEME tool to identify conserved motifs, setting a maximum of 10 motifs with widths ranging from 6 to 50 amino acids. As shown in Figure 5, members with close phylogenetic relationships (e.g., ZmGDS1.7, ZmGDS1.2, ZmGDS1.11, ZmGDS1.5) exhibited highly similar motif compositions and arrangements, including motifs 1, 3, 4, 5, 6, 7, 9, and 2, differing only in the presence of motif 10 at the N-terminus. Another group of closely related members (e.g., ZmGDS1.9, ZmGDS1.4, ZmGDS1.8) also showed similar motif patterns, including an additional motif 8, indicating that motifs 1, 2, 3, 4, 5, 6, 7, and 9 constitute core functional elements of the family. These sequence-level observations are highly consistent with the phylogenetic groupings.

Notably, the motif composition and arrangement of some members were markedly differentiated. ZmGDS1.12 contained only motifs 1 and 5, representing the most simplified structure, suggesting that it may have a distinct function compared to other family members.

### 3.5. Distribution of Cis-Regulatory Elements in ZmGDS1 Gene Promoters

**Figure 6:**
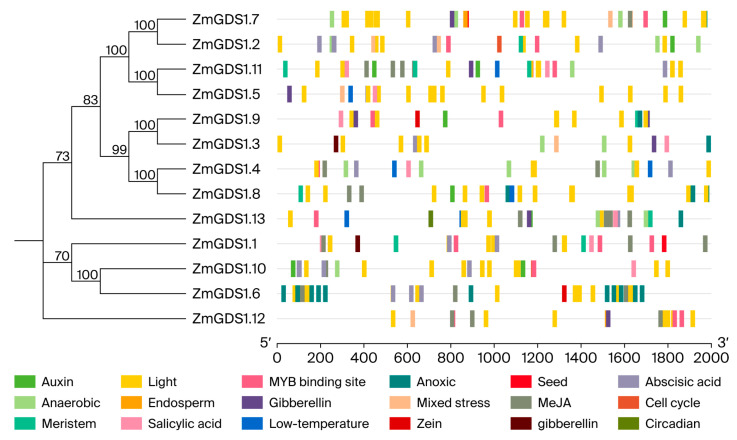
Distribution of cis-acting elements in the promoter regions of *ZmGDS1* family genes. The phylogenetic tree is shown on the left; colored boxes indicate the presence of different element types.

**Figure 7:**
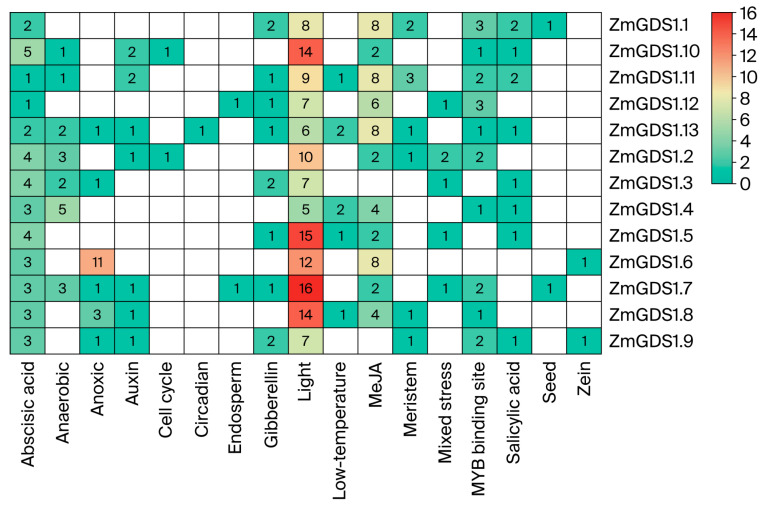
Heatmap depicting the number of cis-acting elements in the promoters of *ZmGDS1* genes. Element categories are color-coded according to functional classification.

To investigate potential regulatory modes of *ZmGDS1* gene expression, we extracted the 2.0 kb upstream sequences from the start codon of each gene as promoter regions and analyzed their cis-acting element composition using PlantCARE. The distribution of elements was then examined in relation to the phylogenetic tree (Figure 6). The number of each element type was quantified and visualized (Table S4) as a heatmap to illustrate enrichment patterns across different *ZmGDS1* genes (Figure 7).

A total of 20 types of cis-acting elements were identified, including those involved in light response, hormone regulation, stress response, tissue-specific development, and transcription factor binding. Light-responsive elements were the most abundant and were present in the promoters of all 13 *ZmGDS1* genes. Among them, *ZmGDS1.7* contained the highest number (16) of light-responsive elements, while *ZmGDS1.5*, *ZmGDS1.10*, and *ZmGDS1.8* also harbored 14 or more such elements, indicating that light responsiveness is a core regulatory feature of the *ZmGDS1* family.

Among hormone-response elements, methyl jasmonate (MeJA)-responsive elements were the most widely distributed, being detected in 12 *ZmGDS1* promoters. Abscisic acid (ABA)- and auxin-responsive elements were found in 10 and 9 promoters, respectively. Gibberellin-responsive elements appeared in only six promoters. Notably, *ZmGDS1.3* contained all four types of hormone-responsive elements (auxin, ABA, MeJA, and gibberellin), suggesting it may serve as a hub integrating multiple hormonal signals.

Regarding stress-responsive elements, hypoxia- and anaerobic-responsive elements were predominant, being present in eight and seven promoters, respectively. Low-temperature-responsive elements were identified in six promoters, while combined stress-responsive elements were found only in *ZmGDS1.2*, *ZmGDS1.3*, and *ZmGDS1.12*. Every *ZmGDS1* promoter contained at least one stress-responsive element. *ZmGDS1.8* and *ZmGDS1.13* each possessed three types of stress-responsive elements (hypoxia, anaerobic, and low temperature), indicating strong potential for stress responsiveness.

In addition, tissue-development-related elements (e.g., meristem, endosperm, seed) and transcription factor binding sites (e.g., MYB binding sites) exhibited significant gene specificity. MYB binding sites were present in 11 *ZmGDS1* promoters, making them the most widely distributed non-environmental response elements. Seed- and zein-specific elements were found exclusively in *ZmGDS1.6*/*ZmGDS1.9* and *ZmGDS1.1*, respectively, suggesting possible specialized roles in seed development.

Phylogenetic analysis revealed that closely related *ZmGDS1* genes (e.g., *ZmGDS1.7*/*ZmGDS1.8*, *ZmGDS1.10*/*ZmGDS1.6*) exhibited highly similar profiles in both the types and numbers of cis-acting elements. In contrast, *ZmGDS1.12*, the most phylogenetically distant member, had the fewest cis-acting elements (12) and lacked most stress- and tissue-development-related elements, reflecting a co-evolution of gene sequence and regulatory mode.

## 4. Discussion

### 4.1. Evolutionary Expansion and Structural Conservation of the ZmGDS1 Family

A total of 13 *GDS1* homologs were identified in the maize genome. This number is similar to that in Arabidopsis (20) and sorghum (15) but lower than in wheat (40) and rice (19), reflecting differential expansion of the *GDS1* family across species. Phylogenetic reconstruction placed the 13 ZmGDS1 proteins into four clades (groups A–D), with group D containing the largest number of maize members (8), indicating lineage-specific expansion within this branch. Chromosomal localization revealed a dispersed distribution across eight chromosomes, including a tandem duplication cluster on chromosome 1 (*ZmGDS1.2/ZmGDS1.3*) and collinear pairs (*ZmGDS1.4/ZmGDS1.8*) that likely arose from whole-genome duplication events. This expansion pattern parallels that of the Arabidopsis *GDS1* family [17] and is consistent with evolutionary trajectories documented for other maize gene families, including the LBD family [22]. These observations suggest that gene duplication has contributed to the expansion and potential functional diversification of the *GDS1* family, possibly facilitating adaptation to diverse developmental and environmental conditions.

### 4.2. Structural Features and Functional Implications of ZmGDS1 Proteins

Conserved domain analysis confirmed that all 13 ZmGDS1 proteins contain the CCCH-type zinc finger domain (PF00642). MEME-based motif identification showed that Motifs 1, 3, and 5 are present in all members; database searches mapped these motifs to the RRM and CCCH core regions, respectively. This structural conservation with AtGDS1 supports the hypothesis that the RNA-binding function of GDS1 is evolutionarily conserved between monocots and dicots. In Arabidopsis, GDS1 localizes to nuclear speckles through its N-terminal RRM and C-terminal RS-like domains and participates in pre-mRNA splicing [23]. The predicted nuclear localization of most ZmGDS1 proteins (Table 2) aligns with such regulatory functions.

Notably, motif composition varies markedly among family members. ZmGDS1.1 contains all 10 identified motifs, consistent with its extended sequence length (716 aa) and complex gene structure (12 exons). In contrast, ZmGDS1.12 harbors only Motifs 1 and 5, representing the most simplified architecture. This structural diversity, also documented in the maize CCCH family [18], points to possible functional specialization, with ZmGDS1.12 potentially fulfilling a distinct role unrelated to stress responses. A subset of members (e.g., ZmGDS1.6, ZmGDS1.10, ZmGDS1.13) exhibited predicted dual or plastid localization (Table 2), suggesting potential functional diversification extending beyond nuclear activities. However, these in silico predictions must be confirmed experimentally.

### 4.3. Promoter Architecture and Regulatory Potential

Promoter cis-element analysis revealed a predominance of light-responsive elements across all *ZmGDS1* genes, with ZmGDS1.7 harboring up to 16 such motifs. This observation is noteworthy given that Arabidopsis GDS1 delays senescence by repressing the light-signaling transcription factors PIF4 and PIF5 [17], raising the possibility that *ZmGDS1* genes may similarly integrate light signals into senescence-regulatory networks.

Hormone- and stress-responsive cis-elements were also prevalent. MeJA-responsive elements appeared in 12 promoters, while ABA- and auxin-responsive elements were detected in 10 and 9 promoters, respectively. Every *ZmGDS1* promoter contained at least one stress-responsive element. The enrichment of hypoxia/anaerobic elements in *ZmGDS1.8* and *ZmGDS1.13* is consistent with the established function of *GDS1* in mediating low-nitrogen stress responses in Arabidopsis [17].

Notably, phylogenetically proximate genes (e.g., *ZmGDS1.7/ZmGDS1.8*, *ZmGDS1.10/ZmGDS1.6*) exhibited comparable cis-element profiles, suggesting coordination between evolutionary relatedness and regulatory architecture—a pattern frequently observed in plant gene family analyses [21,22]. Collectively, these findings point to a potential role for *ZmGDS1* genes in integrating diverse environmental and hormonal signals. It must be emphasized, however, that the presence of cis-elements denotes only potential regulatory sites; their actual functionality requires direct experimental validation.

### 4.4. Hypothesized Roles in Senescence and Future Directions

The results presented here are descriptive and hypothesis-generating. Several lines of evidence lead to testable hypotheses about the potential involvement of *ZmGDS1* genes in low-nitrogen-induced leaf senescence, with possible functional divergence among family members. It is important to note that these hypotheses require experimental validation.

First, ZmGDS1.1 (group A) emerged as the closest homolog to AtGDS1, displaying the most intricate structure and richest motif repertoire. This raises the possibility that it may function as a bona fide ortholog, potentially operating through a dual regulatory mechanism analogous to that described in Arabidopsis—repressing *PIF4/5* under nitrogen-sufficient conditions and undergoing degradation upon nitrogen limitation [17].

Second, the prevalence of stress- and hormone-related cis-elements in *ZmGDS1* promoters points to a potential function in mediating cross-talk between nitrogen availability and senescence-associated signaling pathways. This is particularly evident for *ZmGDS1.3*, which contains all four classes of hormone-responsive elements.

Third, the structural simplicity of ZmGDS1.12 suggests neofunctionalization or subfunctionalization, possibly resulting in a role distinct from stress-induced senescence—perhaps in constitutive RNA processing or tissue-specific development. Such functional diversification within expanded gene families has been well documented in maize [18,22].

This work represents the first systematic characterization of the *GDS1* family in maize, providing a foundational resource for subsequent functional investigations. Several limitations must be acknowledged. Most importantly, all functional inferences presented herein are derived from in silico analyses and remain predictions until validated experimentally.

Future studies should focus on experimental validation using complementary approaches: (1) Profiling *ZmGDS1* expression under varying nitrogen levels by qRT-PCR [24]; (2) confirming subcellular localization with GFP fusion constructs; (3) generating CRISPR/Cas9 knockout lines to assess senescence phenotypes and nitrogen use efficiency; and (4) elucidating molecular networks by identifying interacting proteins and target RNAs [17]. Such investigations will deepen our understanding of maize responses to low-nitrogen stress and may ultimately yield valuable genetic resources for breeding programs aimed at improving nitrogen use efficiency and prolonging leaf functional duration.

## Figures and Tables

**Table 1:** Characterization of *ZmGDS1* genes and ZmGDS1 proteins.

Name	ID	Number of Amino Acid	Molecular Weight	Theoretical pI	Instability Index	Aliphatic Index	Grand Average of Hydropathicity
ZmGDS1.1	Zm00001d033553_T019	716	80,966.24	5.55	55.23	53.49	−0.911
ZmGDS1.2	Zm00001d004651_T002	407	44,970.90	9.10	49.12	48.21	−0.596
ZmGDS1.3	Zm00001d040301_T002	431	46,309.19	8.47	54.21	46.89	−0.529
ZmGDS1.4	Zm00001d040302_T001	471	49,746.34	6.27	55.19	48.34	−0.524
ZmGDS1.5	Zm00001d042363_T005	430	46,825.76	8.76	60.76	45.26	−0.575
ZmGDS1.6	Zm00001d044481_T005	394	43,499.23	6.70	55.74	57.26	−0.552
ZmGDS1.7	Zm00001d049223_T001	443	47,830.83	8.78	52.56	47.25	−0.559
ZmGDS1.8	Zm00001d008322_T003	471	49,727.34	7.22	55.15	48.94	−0.537
ZmGDS1.9	Zm00001d008323_T009	354	38,916.77	7.20	63.33	41.89	−0.703
ZmGDS1.10	Zm00001d011355_T016	355	39,186.78	8.32	55.03	55.86	−0.555
ZmGDS1.11	Zm00001d012049_T002	427	46,348.30	8.68	59.49	46.25	−0.531
ZmGDS1.12	Zm00001d046740_T002	303	31,549.00	9.58	37.98	50.36	−0.532
ZmGDS1.13	Zm00001d023636_T001	522	56,424.51	6.98	53.04	50.33	−0.667

**Table 2:** Predicted subcellular localization of ZmGDS1 proteins.

Name	ID	WoLF PSORT Prediction (Top Three Localizations with Scores)	Plant-mPLoc Prediction
ZmGDS1.1	Zm00001d033553_T019	Nucleus (7.0); Mitochondrion (2.5); Chloroplast (2.0)	Nucleus
ZmGDS1.2	Zm00001d004651_T002	Nucleus (11.0); Chloroplast (1.0); Plasma membrane (1.0)	Nucleus
ZmGDS1.3	Zm00001d040301_T002	Nucleus (13.0); Vacuole (1.0)	Nucleus
ZmGDS1.4	Zm00001d040302_T001	Nucleus (13.0); Vacuole (1.0)	Nucleus
ZmGDS1.5	Zm00001d042363_T005	Nucleus (12.0); Chloroplast (1.0); Mitochondrion (1.0)	Nucleus
ZmGDS1.6	Zm00001d044481_T005	Nucleus (4.0); Chloroplast (3.0); Cytosol (3.0); Mitochondrion (3.0)	Nucleus
ZmGDS1.7	Zm00001d049223_T001	Nucleus (13.0); Chloroplast (1.0)	Nucleus
ZmGDS1.8	Zm00001d008322_T003	Nucleus (13.0); Vacuole (1.0)	Nucleus
ZmGDS1.9	Zm00001d008323_T009	Nucleus (10.0); Chloroplast (1.0); Cytosol (1.0); Plasma membrane (1.0); Vacuole (1.0)	Nucleus
ZmGDS1.10	Zm00001d011355_T016	Nucleus (4.0); Cytosol (4.0); Mitochondrion (3.0)	Nucleus
ZmGDS1.11	Zm00001d012049_T002	Nucleus (11.0); Chloroplast (2.0); Cytosol (1.0)	Nucleus
ZmGDS1.12	Zm00001d046740_T002	Nucleus (7.0); Mitochondrion (3.0); Chloroplast (2.0)	Nucleus
ZmGDS1.13	Zm00001d023636_T001	Chloroplast (4.0); Plasma membrane (3.0); Nucleus (2.5)	Nucleus

## Data Availability

All raw Protein sequences of ZmGDS1 family members and promoter cis-acting element analysis results are included in the Supplementary Information (Datasets Tables S1 and S2). Protein sequences used in this study were retrieved from Phytozome (https://phytozome-next.jgi.doe.gov/ (accessed on 2 March 2026)): Maize (Zm-B73-REFERENCE-NAM-5.0.55), Sorghum (version v3.1.1), Wheat (version IWGSC RefSeq v2.10) and Rice (version v7.0); and TAIR (https://www.arabidopsis.org/ (accessed on 2 March 2026)) for the data on Arabidopsis thaliana. All other data generated or analyzed during this study are available from the corresponding authors (Pengfei Chu, chupengfei@lcu.edu.cn, and Lufei Zhao, lufeizhao@163.com) upon reasonable request.

## Supplementary Materials

The following supporting information can be downloaded at: https://doi.org/10.5281/zenodo.19233060 (20 March 2026), Supplementary Dataset Table S1: Candidate ZmGDS1 protein sequences identified before domain filtering; Supplementary Dataset Table S2: Detailed information of the 10 conserved motifs identified in ZmGDS1 proteins; Supplementary Dataset Table S3: The protein sequences of each species in the phylogenetic evolutionary tree; Supplementary Dataset Table S4: Distribution of cis-acting elements in the *ZmGDS1* gene promoter.
